# Impact of targeted intervention using a collaborative approach for oral third-generation cephalosporins: An interrupted time-series analysis

**DOI:** 10.1017/ash.2022.251

**Published:** 2022-07-11

**Authors:** Ryo Yamaguchi, Koh Okamoto, Takehito Yamamoto, Sohei Harada, Takehiro Tanaka, Hiroshi Suzuki, Kyoji Moriya

**Affiliations:** 1 Department of Pharmacy, the University of Tokyo Hospital, Tokyo, Japan; 2 Department of Infectious Diseases, the University of Tokyo Hospital, Tokyo, Japan; 3 The Education Center for Clinical Pharmacy, Graduate School of Pharmaceutical Sciences, the University of Tokyo, Tokyo, Japan; 4 Department of Infection Control and Prevention, Faculty of Medicine, the University of Tokyo Hospital, Tokyo, Japan

## Abstract

**Objectives:**

To assess the effectiveness of a targeted intervention using a collaborative approach, added to a comprehensive educational intervention, to facilitate the appropriate use of oral third-generation cephalosporins (3GCs).

**Design:**

Quasi-experimental study.

**Setting:**

The University of Tokyo Hospital, a tertiary-care teaching hospital.

**Participants:**

Approximately 2,000,000 outpatients and 80,000 inpatients at the hospital between April 2017 and March 2020.

**Intervention:**

The targeted intervention using the collaborative approach was implemented in the departments with the highest use of oral 3GCs (ophthalmology and dermatology departments). Interrupted time-series analysis was applied to assess the change in days of therapy (DOT) of oral 3GCs between the preintervention period (April 2017–April 2019) and the postintervention period (May 2019–March 2020) for both inpatients and outpatients.

**Results:**

After the introduction of the targeted intervention with oral 3GCs, a significant immediate reduction of 13.48 DOT per 1,000 patient days was detected in inpatients (*P* < .001). However, no significant change in slope was observed before and after the intervention (−0.02 DOT per 1,000 patient days per month; *P* = .94). Although a temporary increase was observed after the targeted intervention in outpatients, the slope significantly decreased (−0.69 DOT per 1,000 outpatient visits per month; *P* = .044). No differences were observed in the use of other oral antibiotics after the intervention.

**Conclusions:**

The targeted intervention contributed to a reduction in DOT of oral 3GCs in both inpatients and outpatients. Targeted interventions using a collaborative approach might be helpful in further decreasing the inappropriate use of antibiotics.

Inappropriate antimicrobial agent use contributes to the increase of antimicrobial resistance (AMR).^1^ Antimicrobial stewardship promotes appropriate use, optimizes treatment efficacy, and minimizes adverse events associated with antibiotic use.^2^ Several antimicrobial stewardship interventions have been proposed and they vary depending on the setting, target prescriber range, target antimicrobials, target infection or syndrome, and approach.^3,4^ Among various interventions, prospective audit and feedback (PAF), also known as handshake stewardship in face-to-face meetings and discussions, is a collaborative approach that has been effective and commonly integrated into intervention implementation.^5^ Although effective, handshake stewardship can be very laborious, which hampers its implementation.^2,6^ Therefore, interventions must be tailored for the available resources and areas requiring improvements at each facility.

The World Health Organization (WHO) reported that oral antibiotics were the most consumed antibiotics (96%–98%) in most countries.^7–9^ The consumption of oral third-generation cephalosporins (3GCs) has been particularly high in Japan compared to European countries or the United States.^10–12^ Oral 3GCs are a class of broad-spectrum antibiotics; however, their role in clinical practice remains limited given their poor bioavailability.^13^ Furthermore, cephalosporins have been associated with increased risk for *Clostridioides difficile* infections (CDI),^14^ and there is concern that their inappropriate use may accelerate the emergence of AMR.^4,15^ Hence, the Japanese government launched the National Action Plan (NAP) on AMR, which included an outcome index requiring that the daily use of oral cephalosporins (including oral 3GCs, quinolones, and macrolides) be reduced per 1,000 inhabitants by 50% in 2020 from the 2013 level.^16^ Consequently, we began comprehensive prescriber education using hospital guidelines on the appropriate use of antibiotics and lectures by an infectious disease (ID) clinical pharmacist on antimicrobial stewardship, targeting inpatient and outpatient oral antibiotic use by all physicians in our hospital in 2017. However, the goal of reducing the use of oral 3GCs was not achieved, indicating that further effective interventions are necessary to further decrease the use of oral 3GCs.

In this study, we adopted a collaborative approach in which we first identified the unmet needs of prescribers and tried to solve their problems and then assessed the effectiveness of our collaborative approach for oral 3GCs combined with comprehensive educational intervention.

## Methods

### Study design and setting

We conducted a quasi-experimental study at the University of Tokyo Hospital (UTH), Tokyo, Japan, between April 2017 and March 2020. UTH is a tertiary-care teaching hospital with 1,226 beds and ∼700,000 annual outpatient visits and ∼27,000 annual inpatient admissions. Physicians can consult ID specialists at any time.

We included all inpatients and outpatients during the study period and investigated the antimicrobial consumption by department. To assess the impact of the targeted intervention for oral 3GC consumption, antibiotic use before (phase 1: April 2017–April 2019) and after the targeted intervention (phase 2: May 2019–March 2020) was compared using interrupted time-series (ITS) analysis (Appendix 1). The available oral 3GCs at UTH included cefcapene-pivoxil (CFPN-PI), cefditoren-pivoxil, cefpodoxime-proxetil, and cefdinir. The Institutional Review Board and Ethics Committee of the Graduate School of Medicine and Faculty of Medicine, University of Tokyo, approved the study protocol (approval no. 2020171NI, 2529) and waived the need for patients’ written informed consent. This study was conducted according to the ethical standards specified in the 1964 Declaration of Helsinki and its later amendments.

### Phase 1 (April 2017–April 2019): Comprehensive intervention for all oral antibiotics

At UTH, a multidisciplinary antimicrobial stewardship team (AST) was established in April 2015 to promote the appropriate use of antibiotics by implementing programs for the entire hospital. The AST, consisting of ID specialists, clinical pharmacists, and clinical microbiologists, has been performing daily PAF since April 2015 for patients treated with anti–methicillin-resistant *Staphylococcus aureus* agents or carbapenems.

The AST commenced comprehensive interventions for oral antibiotics usage in June 2017 (1) by incorporating the government guidelines, where antibiotics are mostly not indicated for common cold and gastrointestinal infections, into the institutional guidelines; (2) by sending an annual usage report on oral cephalosporins, quinolones, and macrolides for each department to all clinical departments; and (3) by providing online learning materials for all medical staff (Table 1).

**Table 1.** Description of Comprehensive Intervention for Oral Antibiotics

Date	Comprehensive INTERVENTION
June 2017	Distributing the antimicrobial stewardship manual (1st edition, digest version) published by the Government of Japan Ministry of Health, Labor, and Welfare and posting the National Action Plan on AMR in Japan (1st edition) on the institution’s website.
July 2017	Reporting the annual results of oral antimicrobial consumption, focusing on oral cephalosporins, quinolones, and macrolides use in each department and promoting appropriate use
Oct 2017	Lecture by ID specialist and clinical pharmacist on antimicrobial stewardshipfor all staff
June 2018	Major revisions of the hospital guideline on appropriate use of antibiotics
July 2018	Lecture by ID clinical pharmacist about the National Action Plan on AMR in Japan to each representative staff
	Request for review of the order sets and clinical pathways for antimicrobial use
Sept 2018	Lecture by ID specialist and clinical pharmacist on antimicrobial stewardshipfor all staff
Dec 2018	All healthcare professionals’ e-learning on oral antibiotic prescribing focusing on the National Action Plan on AMR
Feb 2019	All healthcare professionals’ e-learning on appropriate use of oral antibiotics (eg, notes on the use of oral antimicrobials including bioavailability)
July 2019	Notification of annual trends in the use of 3 oral antimicrobial classes (cephalosporins, quinolones, and macrolides) in our hospital and a request for appropriate use of perioperative antimicrobial agents
	Minor revisions on hospital guideline for appropriate use of antibiotics
	Lecture by ID clinical pharmacist about the national action plan on AMR in Japan for each representative physician
Dec 2019	All healthcare professionals’ e-learning on oral antibiotic prescribing focusing on the national action plan on AMR
Feb 2020	All healthcare professionals’ e-learning on appropriate use of oral antibiotics (eg, national action plan on AMR, bioavailability of oral antibiotics),

Note. AMR, antimicrobial resistance; ID, infectious disease.

### Phase 2 (May 2019– March 2020): Targeted intervention for oral 3GCs

In phase 1, we confirmed that the comprehensive intervention reduced the annual 3GC use by 11% among inpatients and 17% among outpatients in 2017 compared to 2016, although the Japanese NAP goal of 50% reduction was not achieved. The departments of ophthalmology (inpatients) and dermatology (outpatients) were identified as the top oral 3GC users; therefore in 2017, the following tailored interventions were executed to facilitate further reduction of oral 3GC use.

For the ophthalmology department, we audited oral 3GC use in inpatients and found that CFPN-PI was routinely prescribed for 3 days (from the day of surgery) after cataract surgery. Although perioperative oral antibiotics, including CFPN-PI, have been used in Japan for prophylaxis against various infections, including ocular infections, data on its clinical efficacy and aqueous humor penetration were lacking.^17^ After multiple discussions with the ophthalmology department, we conducted clinical research in collaboration with them to evaluate the aqueous humor penetration of orally administered CFPN-PI. The results showed that oral CFPN-PI poorly penetrated the aqueous humor.^18^ Because CFPN-PI is very unlikely to be effective in preventing endophthalmitis after cataract surgery, it was removed from the order set by April 2019 (without any alternative antibiotics).

For the dermatology department, we provided a one-time face-to-face lecture in April 2019. To ensure effectiveness, we first had discussions with an attending dermatologist in charge of clinical care on how to best deliver our message to all the members in the dermatology department with various levels of experience. According to the advice from the attending dermatologist, we asked the dermatologists (∼30 physicians, including attending physicians and physicians in training) to complete a questionnaire to provide information on their understanding and raise questions on the use of oral 3GCs and other antibiotics in their field. We then provided a face-to-face lecture specifically addressing their questions and concerns. The lecture covered the background, strategies against AMR, AMR action plan in Japan, appropriate use of antibiotics in dermatology, prevention of surgical site infections, and remedial education on UTH guidelines for appropriate antibiotic use. During the lecture, questions were encouraged, raised, and answered. We received questions even after the lecture, which we answered.

### Outcome

The primary outcome was days of therapy (DOT) of oral 3GCs in inpatients and outpatients. For inpatients, the amount of antibiotics used was evaluated by recording the DOT per 1,000 patient days (DOT/1,000 PD).^19^ For outpatient, the total number of outpatient visits per month was the denominator, and DOT per 1,000 outpatient visits (DOT/1,000 OV) were calculated according to previous reports.^20^ Antibiotics were classified according to the WHO Anatomical Therapeutic Chemical classification system.^21^ Secondary outcomes included (1) DOT of other oral antibiotics (penicillins, other cephalosporins, fluoroquinolones, and macrolides) in inpatients and outpatients; (2) DOT of intravenous antibiotics; and (3) incidence of hospital-acquired CDI (HA-CDI) calculated as the number of inpatients with laboratory-confirmed *C. difficile* toxin production ≥72 h after hospital admission. Patients with multiple *C. difficile* toxin-production detections were only counted once. We confirmed *C. difficile* toxin production using the GE immunochromato-CD GDH/TOX test (Nissui, Tokyo, Japan).

### Statistical analysis

The Mann-Whitney *U* test was used for continuous variables. Details of the ITS analysis are described in Appendix 1. All statistical analyses were performed using IBM SPSS Statistics version 24 software (IBM, Armonk, NY), and statistical significance was set at *P* < .05.

## Results

During the study period, there were ∼2,000,000 outpatient visits and ∼80,000 hospital admissions, with no major changes in the number of outpatient visits, hospital admissions, and length of hospital stay. Data on the overall incidence of surgical site infections or complications from surgery were unavailable.

### Trends in oral 3GC use

**Fig 1.**
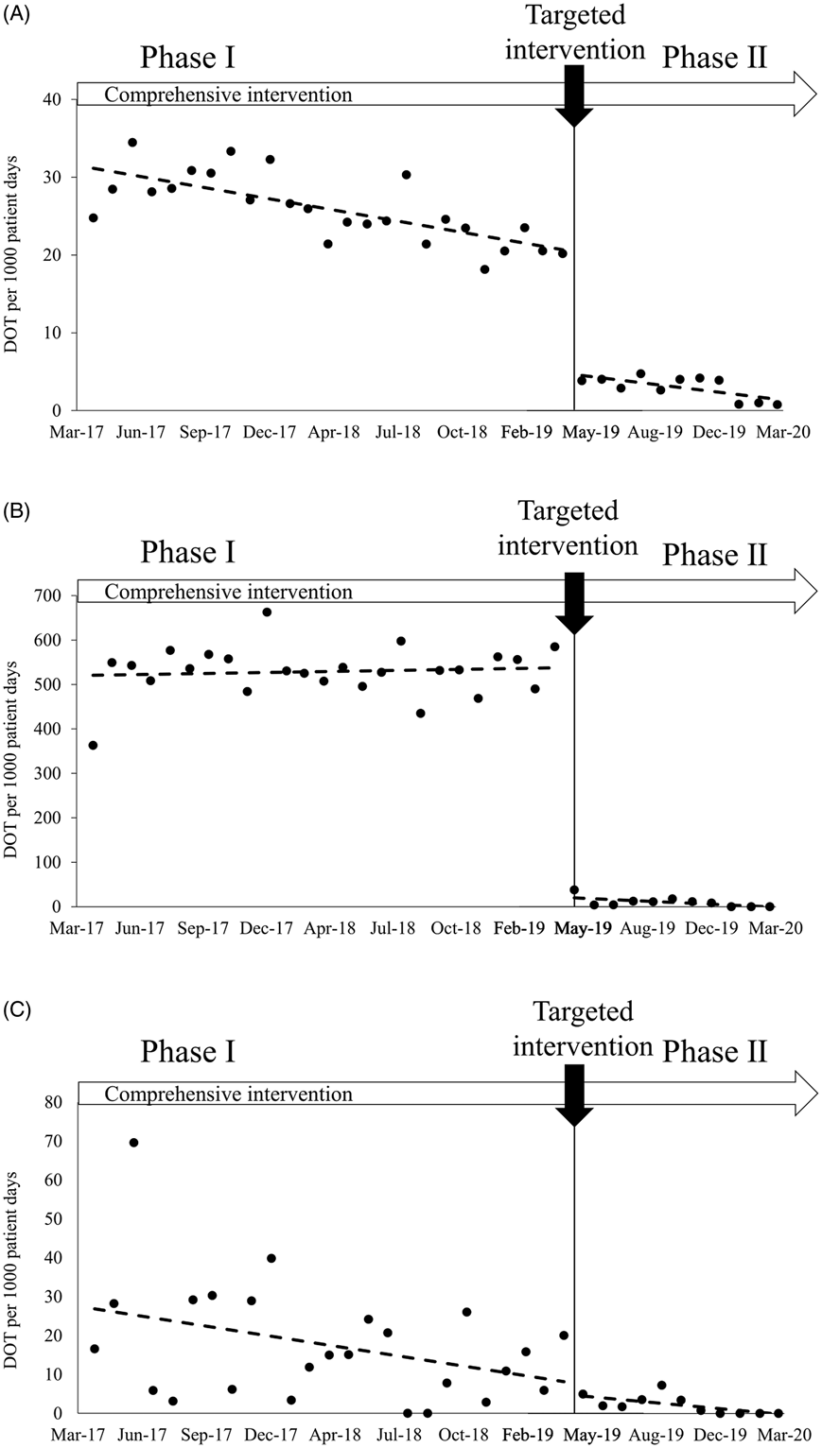
Segmented linear regression of oral third-generation cephalosporins (3GCs) days of therapy (DOT) per 1000 patient days in inpatients, before and after the targeted intervention. (A) All departments. (B) Ophthalmology. (C) Dermatology.

Regarding DOT/1,000 PD for oral 3GCs in inpatients, there was a statistically significant decrease in the trend by 0.44 DOT/1,000 PD/month (*P* < .001) in phase 1 for all departments (Fig. 1A and Table 2). After the targeted intervention, there was a significant immediate reduction by 13.48 DOT/1,000 PD (*P* < .001). However, no significant change in slope was observed between the 2 phases. In the ophthalmology department, there was no significant trend change during phase 1 (Fig. 1B and Table 2). After the targeted interventions, there was a statistically significant immediate reduction (−518.32 DOT/1,000 PD; *P* < .001), with the ophthalmology department accounting for ∼80% of the reduction. However, there was no significant change in the slope. For the dermatology department, there was a significant decreasing trend (−0.78 DOT/1,000 PD/month; *P* = .035) during phase 1 (Fig. 1C and Table 2). After the targeted intervention, there were no significant changes in both levels and slope between the 2 phases.

**Table 2.** Interrupted Time-Series Analysis of Oral Third-Generation Cephalosporins for Inpatients in 3 Groups

Group	Trend During Phase 1; *β*_1_ (95% CI)	*P* Value	Change in Level; *β*_2_ (95% CI)	*P* Value	Change in Slope Between the 2 Phases; *β*_3_ (95% CI)	*P* Value
All	−0.44 (−0.59 to −0.28)	<.001	−13.48 (−17.43 to −9.53)	<.001	−0.02 (−0.58 to 0.54)	.94
Ophthalmology	0.70 (−2.10 to 3.50)	.62	−518.32 (−588.81 to −447.82)	<.001	−2.73 (−12.8 to 7.29)	.58
Dermatology	−0.78 (−1.51 to −0.06)	.035	6.63 (−11.59 to 24.85)	.46	−0.67 (−3.26 to 1.92)	.60

Note. CI, confidence interval.

**Fig 2.**
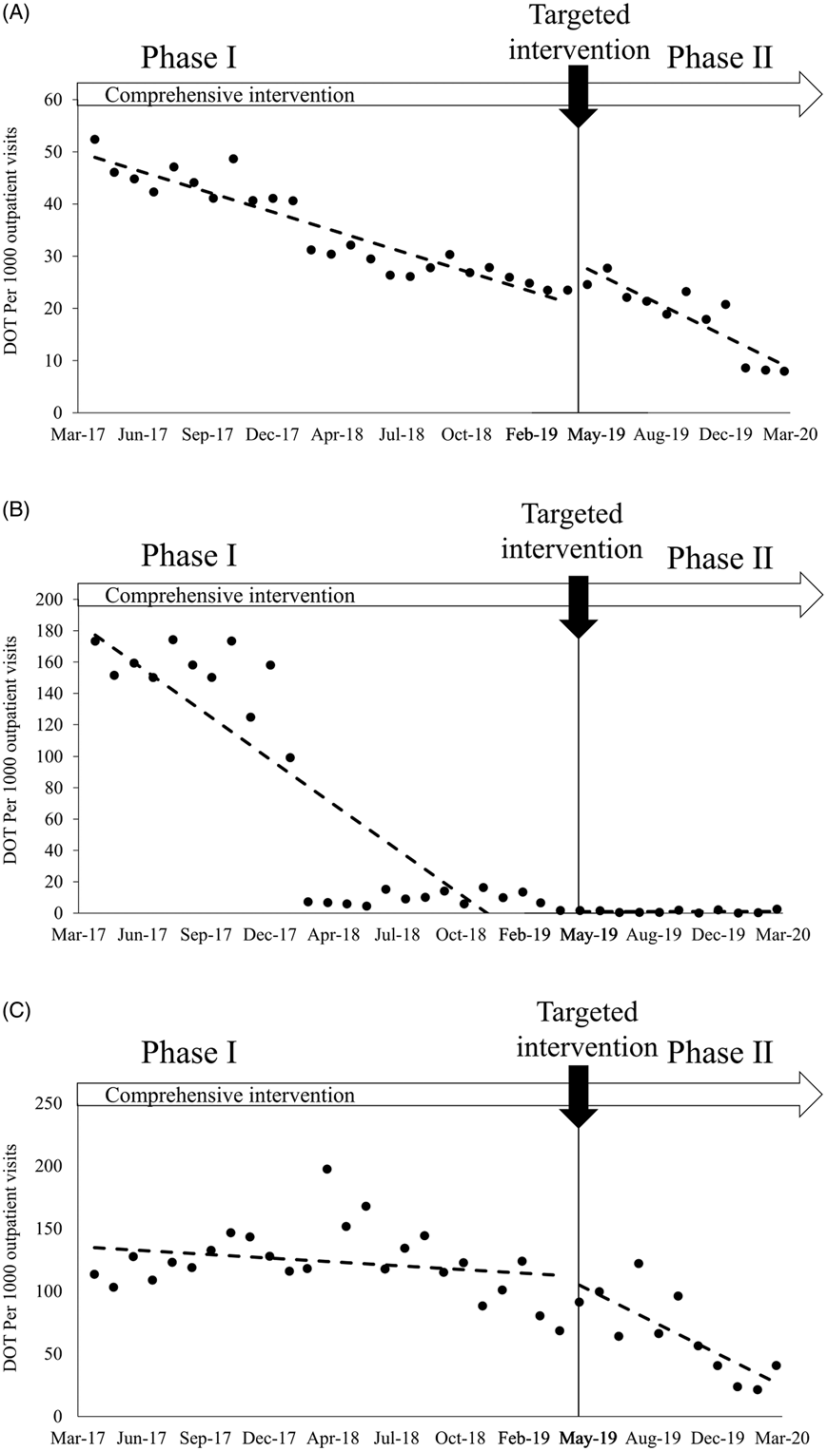
Segmented linear regression of oral third-generation cephalosporins (3GCs) days of therapy (DOT) per 1,000 outpatient visits among outpatients, before and after the targeted intervention. (A) All departments. (B) Ophthalmology. (C) Dermatology.

Regarding DOT/1,000 OV for oral 3GCs in outpatients, there was a statistically significant decreasing trend (−1.16 DOT/1,000 OV/month; *P* < .001) during phase 1 for all departments (Fig. 2A and Table 3). After the targeted intervention, an immediate increase was observed (7.67 DOT/1,000 OV; *P* = .002), and the slope significantly decreased (−0.69 DOT/1,000 OV/month; *P* = .044). For the ophthalmology department, there was a significant decrease in trend (−8.78 DOT/1,000 OV/month; *P* < .001) during phase 1 (Fig. 2B and Table 3). For the dermatology department, there was no significant decrease in trend during phase 1 (Fig. 2C and Table 3). After the targeted intervention, there was no significant change in levels, but the slope significantly decreased (−6.90 DOT/1,000 OV/month; *P* = .01).

**Table 3.** Interrupted Time-Series Analysis of Oral Third-Generation Cephalosporins for Outpatients in 3 Groups

Group	Trend During Phase 1; *β*_1_ (95% CI)	*P* Value	Change in Level; *β*_2_ (95% CI)	*P* Value	Change in Slope Between 2 Phases; *β*_3_ (95% CI)	*P* Value
All	−1.16 (−1.35 to −0.97)	<.001	7.67 (2.94 to 12.39)	.002	−0.69 (−1.36 to −0.02)	.044
Ophthalmology	−8.78 (−10.55 to −7.02)	<.001	45.48 (1.12 to 89.83)	.045	8.82 (2.52 to 15.13)	.008
Dermatology	−0.92 (−2.35 to 0.51)	.20	−6.94 (−43.01 to 29.14)	.70	−6.90 (−12.02 to −1.77)	.01

Note. CI, confidence interval.

Contrary to the observed decreases in oral 3GC use, no statistically significant increases were observed in the use of other oral or intravenous antibiotics (Table 4).

**Table 4.** Interrupted Time-Series Analysis for Hospital-wide Antimicrobial Consumption

Antibiotics	Inpatient						Outpatient					
	Trend During Phase 1; *β*_1_ (95% CI)	*P* Value	Change in Level; *β*_2_ (95% CI)	*P* Value	Change in Slope Between 2 Phases; *β*_3_ (95% CI)	*P* Value	Trend During Phase 1; *β*_1_ (95% CI)	*P* Value	Change in Level; *β*_2_ (95% CI)	*P* Value	Change in Slope Between 2 Phases; *β*_3_ (95% CI)	*P* Value
1GC	0.05 (−0.14 to 0.23)	.62	−0.17 (−4.76 to 4.42)	.94	0.50 (−0.16 to 1.15)	.13	0.44 (0.26 to 0.63)	<.001	−1.21 (−5.83 to 3.41)	.60	0.30 (−0.36 to 0.95)	.36
Fluoroquinolones	0.32 (−0.05 to 0.68)	.091	1.98 (−7.3 to 11.25)	.67	−0.59 (−1.91 to 0.73)	.37	0.23 (−0.01 to 0.46)	.058	0.09 (−5.79 to 5.96)	.98	0.40 (−0.43 to 1.24)	.33
Macrolides	0.22 (0.01 to 0.43)	.037	−2.42 (−7.63 to 2.79)	.35	−0.12 (−0.86 to 0.62)	.73	0.66 (−0.06 to 1.38)	.073	−7.70 (−25.86 to 10.46)	.39	0.26 (−2.32 to 2.84)	.84
Penicillins	0.04 (−0.2 to 0.28)	.76	5.79 (−0.28 to 11.86)	.061	−0.47 (−1.33 to 0.39)	.28	0.49 (0.17 to 0.82)	.004	6.25 (−1.97 to 14.47)	.13	0.62 (−0.55 to 1.79)	.29
IV antibiotics	0.10 (0.04 to 0.16	.002	0.93 (−0.55 to 2.41)	.21	−0.22 (−0.43 to −0.01)	.044	…	…	…	…	…	…

Note. 1GC, first-generation cephalosporins; CI, confidence interval; IV, intravenous.

### CDI

HA-CDI incidence was not statistically different between phases 1 and 2 (0.34 vs 0.33 per 1,000 PD; *P* = .19).

## Discussion

We evaluated the effectiveness of targeted intervention using a collaborative approach for oral 3GCs by assessing time-series changes in antimicrobial use before and after the intervention. We detected a significant immediate reduction in 3GC use in inpatients, and the slope significantly decreased in outpatients after the targeted intervention. We achieved a 50% reduction in the DOT of oral 3GCs, the goal of Japanese NAP; however, the use of other oral antibiotics and HA-CDI incidence remained largely unchanged.

In the present study, a hospital-wide educational intervention was provided to all prescribers of oral antimicrobials, focusing on 3 antimicrobial classes (cephalosporins, quinolones, and macrolides) in phase 1. Although a certain degree of reduction was observed, the target was not achieved. Previous reports showed that educational interventions optimized antimicrobial use, but this intervention alone was inadequate. Thus, unidirectional education, such as lectures or informational pamphlets, should be used to complement other stewardship activities.^22,23^ Therefore, we added an intervention targeted at a specific department using a collaborative approach in phase 2. It was a behavioral science-based intervention^24^ with a collaborative approach to support prescriber behavioral change and resulted in decreased oral 3GC use (Tables 2 and 3). Furthermore, the results of the sensitivity analyses (Appendix 2 and Supplementary Table 1) revealed that the targeted intervention to the ophthalmology and dermatology departments affected the hospital-wide use of oral 3GCs in inpatients and outpatients, respectively. Uda et al^20^ reported that educational intervention against inappropriate oral 3GC use decreased DOT without affecting patients’ clinical outcomes. However, to the best of our knowledge, this is the first report to show that the addition of a department-specific targeted collaborative approach to hospital-wide education further reduces oral 3GC use.

There are several barriers to changing physicians’ antimicrobial prescription behaviors. First, basic education on the appropriate use of antimicrobial agents tends to be conceptual (eg, “as narrow as possible”) and unidirectional. Therefore, it is difficult for clinicians to view it as knowledge that is readily applicable to daily practice. Second, physicians often dismiss the long-term benefit of the appropriate use of antimicrobials, that is, the prevention of the development of bacterial resistance.^25^ Third, physicians’ knowledge outside their expertise often remains outdated. Therefore, it may be difficult to encourage physicians to change their individual behavior through unidirectional education alone. Hence, we hypothesized that addressing their unique problems and needs and solving them collaboratively can have a crucial impact. In our study, analysis of the prescribing patterns and questionnaires revealed routine order sets in the ophthalmology department and a lack of knowledge on the common causative organisms of skin infections.

In the dermatology department, the corresponding spectrum of antimicrobial agents was the main driver for the physicians’ prescribing patterns. Therefore, we provided a one-time face-to-face lecture for dermatologists to resolve knowledge gaps and follow-up after the lecture, which may have led to a reduction in the consumption of oral 3GCs in the dermatology department. Interestingly, time profiles of DOT in inpatients and outpatients in the dermatology department showed different patterns: a significant decreasing trend during phase 1 and nonsignificant change in slope between phases 1 and 2 in inpatients (Fig. 1C), and a nonsignificant decreasing trend in phase 1 and a significant change in slope between phases 1 and 2 in outpatients (Fig 2C). This observation may be explained by the difference in the physicians’ experience between inpatients and outpatients. We hypothesize that the intervention provided in phase 1 focusing on general issues was effective enough to reduce inappropriate prescription because many junior physicians (including residents) directly care for inpatients. However, the targeted intervention, which was relatively advanced in contents, may have been less effective for junior physicians, and, consequently, no significant slope change was observed between phases 1 and 2. Conversely, since senior physicians directly care for outpatients, most of them already understood the contents of the intervention in phase 1; thus, no significant downward trend was observed in phase 1. In contrast, the contents of the targeted intervention were informative even to senior physicians, and this may have led to the significant change in slope between phases 1 and 2.

We did not ask or force the ophthalmology department to remove the oral 3GCs from the order set; instead, we had an open discussion with them to revisit the role of prophylaxis with oral 3GCs, which led to a collaborative clinical study.^18^ Based on this study’s results, the ophthalmology department removed CFPN-PI from the prophylaxis regimen. Several studies reported that interventions focusing on facility-specific order sets and guidelines can optimize antimicrobial use.^2,26,27^ A review of the order set targeting specific departments with high use of antibiotics may bring about a high return because a collaborative approach can easily be adopted without much human resource. Additionally, the completion of a questionnaire to identify areas of input by prescribers may help foster more effective education. Such multifaceted educational methods contribute to the reduction of antimicrobial use.^28^ Our findings reinforce the importance of addressing the barriers specific to individual groups and tailoring interventions accordingly. Although we focused on oral 3GCs, the findings of this study may apply to other antibiotics and settings.

Notably, inpatients and outpatients showed different time profiles of DOT of oral 3GCs; especially, a statistically significant immediate increase in level was observed only in outpatients (7.67 DOT/1,000 PD; *P* = .002) (Table 3). Because no immediate increase in levels was observed in either the ophthalmology or the dermatology department (Fig. 2 and Table 3), it is unlikely that targeted interventions for these departments directly affected the observed immediate increase in levels in all departments. During the study periods, only general interventions were implemented in all departments other than the ophthalmology and dermatology departments. Additionally, the timing of the targeted interventions in the ophthalmology and dermatology departments overlapped with the timing of physician turnover at UTH when the educational effects of the general interventions are reset. Therefore, antimicrobial use may have been temporarily inappropriate at this time in all departments other than the ophthalmology and dermatology departments, and, consequently, a significant immediate increase in levels may have been observed.

Previous studies showed that the implementation of antimicrobial stewardship significantly reduced the incidence of HA-CDI in hospitals.^29,30^ However, we observed no significant reduction in the incidence of HA-CDI after the targeted intervention. This is clinically plausible because the baseline incidence of HA-CDI in our hospital was lower than that in other Japanese hospitals.^31^ Furthermore, the incidence of CDI is influenced by various factors other than antimicrobial use, such as adherence to infection control behaviors.^32^ Moreover, the use of other antibiotics in relation to HA-CDI, such as the second- and fourth-generation cephalosporins, penicillins, and fluoroquinolones, did not change.^33^ Additional interventions are needed to further decrease HA-CDI.

This study had several limitations. We did noy employ a control group, and the ITS analysis design did not allow us to rule out confounding by cointerventions or other events that occurred during the intervention. However, to the best of our knowledge, there was no major change in practice or patient population that could have directly affected oral antibiotic prescription at UTH during the study period. By the end of the study period, coronavirus disease 2019 (COVID-19) had not spread widely in Japan, and UTH cared for only 1 patient with COVID-19. Therefore, the COVID-19 pandemic appears to have had little impact on antibiotic use at UTH during the study period. This study was conducted at a single center in Japan and it had a before-and-after comparative design. Thus, caution is required when interpreting our findings. Because the problems related to inappropriate antimicrobial use vary between facilities, individualized approaches are needed. However, as discussed above, targeted interventions using a collaborative approach may work well in other facilities and may be viable options to further decrease the use of specific antibiotics of concern. The long-term sustainability of the effects of our interventions was not assessed. The effect of education may gradually wane because of physician turnover; thus, regular interventions might be necessary for the long-term suppression of inappropriate antibiotic use. The drivers of the observed changes, perhaps through a qualitative study in the future should be further evaluated.

In conclusion, our study demonstrated the usefulness of targeted interventions using a collaborative approach against oral 3GCs combined with comprehensive interventions. We believe that targeted interventions using a collaborative approach may be helpful in further decreasing the inappropriate use of antibiotics.
